# Androgen receptor expression reduces stemness characteristics of prostate cancer cells (PC3) by repression of CD44 and SOX2

**DOI:** 10.1002/jcb.27573

**Published:** 2018-09-11

**Authors:** Deepa Srinivasan, Linda Senbanjo, Sunipa Majumdar, Renty B. Franklin, Meenakshi A. Chellaiah

**Affiliations:** ^1^ Department of Oncology and Diagnostic Sciences University of Maryland Dental School Baltimore Maryland

**Keywords:** androgen receptor, cancer stem cells, cluster of differentiation 44, prostate cancer, sex‐determining region Y (SRY)‐box 2

## Abstract

Studies have shown that a subgroup of tumor cells possess stemness characteristics having self‐renewal capacity and the ability to form new tumors. We sought to identify the plausible stemness factor that determines the “molecular signature” of prostate cancer (PCa) cells derived from different metastases (PC3, PCa2b, LNCaP, and DU145) and whether androgen receptor (AR) influences the maintenance of stemness features. Here we show sex‐determining region Y (SRY)‐box 2 (SOX2) as a putative stem cell marker in PC3 PCa cells and not in DU145, PCa2b, or LNCaP cells. PCa2b and PC3 cells were derived from bone metastases. PCa2b cells which are positive for the AR failed to demonstrate the expression of either cluster of differentiation 44 (CD44) or SOX2. Knockdown (KD) of AR in these cells did not affect the expression of either CD44 or SOX2. Conversely, PC3 cells, which are negative for AR, expressed both CD44 and SOX2. However, the expression of AR downregulated the expression of both CD44 and SOX2 in PC3 cells. CD44 regulates SOX2 expression as KD of CD44 and reduces SOX2 levels considerably. SOX2 KD attenuated not only the expression of SNAIL and SLUG but also the migration and tumorsphere formation in PC3 cells. Collectively, our findings underscore a novel role of CD44 signaling in the maintenance of stemness and progression of cancer through SOX2 in AR‐independent PC3 cells. SOX2 has a role in the regulation of expression of SNAIL and SLUG. SOX2 could be a potential therapeutic target to thwart the progression of SOX2‐positive cancer cells or recurrence of androgen‐independent PCa.

**Abbreviations**
AR: androgen receptor
CD44: cluster of differentiation 44
CSC: cancer stem cells
EMT: epithelial‐mesenchymal transition
IB: immunoblot
OCT4: octamer‐binding transcription factor 4
PCa: prostate cancer
RT‐PCR: reverse‐transcription polymerase chain reaction
siRNA: small interfering RNA
SOX2: sex‐determining region Y (SRY)‐Box 2

## 1 INTRODUCTION

Prostate cancer (PCa) is the second leading cause of cancer‐related death among men in the United States, behind only lung cancer.^1^ It is a disease of extensive metastases with secondary lesions commonly occurring in lymph nodes, brain, bones, and sometimes in visceral organs such as the liver and lungs.^2^, ^3^ While androgen‐deprivation therapy targeted toward androgen receptor (AR) signaling is widely used for advanced PCa, this therapeutic strategy has been marred by major clinical limitations. Patients who initially respond to androgen‐deprivation therapy progress to develop an androgen independent stage or castration‐resistant PCa, which is unresponsive to hormone deprivation resulting in relapse with a more aggressive disease.^4^, ^5^

Emerging evidence from various human tumors indicates the presence of heterogeneous populations of cells within tumor bulk that comprise tumor cells and a small subpopulation of somatic cells with stem‐like properties, termed as cancer stem cells (CSCs).^6^, ^7^ CSCs are considered to be major drivers of treatment failure and tumor recurrence due to their ability to resist and survive through conventional therapies and their dynamic self‐renewal capabilities. Recent reports indicate the potential expansion of CSCs triggered by selection pressures imposed by androgen‐deprivation therapy in PCa.^8^, ^9^, ^10^ Therefore, we aim to understand the association between the CSC phenotype and AR. One of the potential mechanistic explanations put forward involves the ability of the AR to regulate sex‐determining region Y (SRY)‐box 2 (SOX2), octamer‐binding transcription factor 4 (OCT4), and NANOG transcription factors. These factors constitute the core embryonic transcription factor machinery.^11^, ^12^, ^13^ Pivotal work by Takahashi et al^14^ showed that SOX2 and OCT4, together with KLF4 and c‐MYC could reprogram human somatic cells to pluripotent stem cells.^15^ The ability of these factors to confer stemness would have major clinical implications, whereby their re‐expression in adult CSCs could result in a more aggressive tumor phenotype. Dysregulated expression of these factors has been widely associated with enhanced tumorigenicity.^11^, ^16^, ^17^ For instance, SOX2 overexpression was found to promote extensive metastasis of breast and PCa cells through epithelial‐mesenchymal transition (EMT).^18^, ^19^, ^20^

EMT is one of the key steps in the metastatic process whereby epithelial cells lose cell‐cell contact and polarity, becoming more migratory and invasive. EMT demonstrates the expression of transcriptional factors (eg, SNAIL and SLUG), downregulation of the epithelial marker (eg, E‐cadherin), and upregulation of mesenchymal markers (eg, N‐cadherin, vimentin, and fibronectin).^21^, ^22^, ^23^ Cancer cells that undergo EMT are thought to acquire CSC‐like features, making them more compatible for metastasis.^9^, ^10^ Studies in pancreatic ductal adenocarcinoma and colorectal cancer have interrelated CSC phenotype and EMT, and have implicated the core embryonic transcription factors to be critical in mediating their shared properties.^24^, ^25^ Interestingly, in PCa the androgen deprivation therapeutic approach has been shown to promote the acquisition of EMT phenotype.^26^ Because androgen deprivation therapy also results in the expansion of CSCs, these findings suggest that a potential AR‐CSC‐EMT axis may be responsible for advanced PCa progression.

Cluster of differentiation 44 (CD44) is a transmembrane receptor that is known to have significant roles in cell‐cell interactions, cell adhesion, and migration.^27^ CD44 signaling via extracellular matrix ligands such as hyaluronic acid, osteopontin, collagens, and matrix metalloproteinases has been shown to increase the metastatic potential of cancer cells.^28^ Moreover, CD44 has also been recognized as a marker of CSCs in breast and PCa.^28^, ^29^

Our current study aims to characterize the expression patterns of stemness factors SOX2, OCT4, and NANOG in PCa cell lines derived from different metastases, to identify the key stemness factor that determines the “molecular signature” of potential PCa stem cells. Additionally, we also wanted to examine whether AR has a role in the maintenance of stemness characteristics of these PCa cells. Strikingly, we show here, (a) the antagonistic effect of AR in the expression of SOX2 and CD44; and (b) the vital role of CD44‐SOX2 signaling axis in the expression of SNAIL and SLUG, as well as cell migration.

## 2 MATERIALS AND METHODS

### 2.1 Cell culture

PC3 cell line stably expressing AR (PC3/AR^+^) was generated in Dr Renty B. Franklin’s Laboratory (University of Maryland Dental School, Baltimore, MD). Stable PC3 cell line knockdown (KD) of CD44 (PC3/CD44^−^) was generated as described previously^30^. PC3, LNCaP, DU145, PC3/AR^+^, and PC3/CD44^−^ cells were cultured in Roswell Park Memorial Institute (RPMI)‐1640 medium containing 10% fetal bovine serum (FBS), as previously described.^3^, ^30^ Also, normal prostatic epithelial cells (HPR1), which are used as controls were cultured in keratinocyte medium supplemented with epidermal growth factor (EGF) (2.5 mg/500 mL) and bovine pituitary extracts (25 mg/500 mL) (Gibco, Life Technologies, Bethesda, MD).^31^ MDA PCa2b cells (referred to as PCa2b) were cultured in BRFF‐HPC1 (AthenaES, Baltimore, MD) medium containing only 10% FBS, slight modifications from previously described specifications.^32^ The FBS used in PCa2b culture media is nonheat inactivated (16000036; Gibco, Life Technologies). All cell culture media were supplemented with penicillin and streptomycin (1%) and the cells were maintained at 37°C in a humidified incubator with 5% CO_2_.

### 2.2 Reverse‐transcription polymerase chain reaction

Reverse‐transcription polymerase chain reaction (RT‐PCR) was done as described previously.^33^ Briefly, total RNA was extracted from different cell lines using the RNeasy Midi kit (Qiagen, Valencia, CA). Complementary DNA (cDNA) was synthesized from RNA using SuperScript III First‐Strand Synthesis System (Invitrogen, Carlsbad, CA), with 5 μg of total RNA. RT‐PCR for NANOG, OCT4, SOX2, and glyceraldehyde 3‐phosphate dehydrogenase (GAPDH) was done with the cDNA generated, JumpStart REDTaq ReadyMix PCR Reaction Mix (Sigma‐Aldrich, St Louis, MO), and the appropriate primer sets. GAPDH level was used for normalization. Samples were electrophoresed on 1.5% agarose gel and stained with GelGreen (Biotium Inc, Hayward, CA). Primers (forward [F] and reverse [R]) and the expected product size (in base pairs, bp) are provided below:

NANOG (470 bp): F: 5′‐ACCTATGCCTGTGATTTGTGG‐3′,
R: 5′‐AAGAGTAGAGGCTGGGGTA GG‐3;
OCT4 (470 bp): F: 5′‐GAGAATTTGTTCCTGCAGTGC‐3′,
R: 5′‐GTTCCCAATTCCTTCCTTAGT G‐3;
SOX2 (424 bp): F: 5′‐ATGGGTTCGGTGGTCAAGTC‐3′,
R: 5′‐GTGGATG GGATTGGTGTTCTC‐3′;
GAPDH (452 bp): F: 5′‐ACCACAGTCCATGCCATCAC‐3′,
R: 5′‐TCCACCACCCTGTTGCTGTA‐3′.

### 2.3 RNA extraction and quantitative real‐time PCR

Total RNA was extracted from PC3 cells with SOX2 KD and control cells using RNeasy Midi Kit (Qiagen). cDNA was synthesized from RNA using SuperScript III First‐Strand Synthesis System (Invitrogen), with 3 μg of total RNA. The cDNA generated was used for standard real‐time PCR analysis to quantitate levels of SOX2, OCT4, NANOG, CD44, and GAPDH transcripts using custom primers and SYBR Universal Master Mix (Applied Biosystems, Foster City, CA). Each reaction was performed in triplicates in 20 μL volume in 96‐well plates in an ABI 7000HT thermocycler (Thermo Fisher Scientific, Waltham, MA) (2 minutes at 50°C, 10 minutes at 95°C, 40 cycles for 15 seconds at 95°C, and 1 minute at 60°C). The gene expression was calculated relative to that of control cells and normalized for GAPDH and measured under the same conditions using the $2^{-\Delta \Delta C_{t}}$ method.^30^, ^34^

The forward (F) and reverse (R) primers used for the genes are as follows:

NANOG: F: 5′‐ATGCCTCACACGGAGACTGT‐3′,
R: 5′‐AAGTGGGTTGTTT GCCTTTG‐3′;
OCT4: F: 5′‐TCGAGAACCGAGTGAGAGG‐3′,
R: 5′‐GAACCACACTCGGACCACA‐3′;
SOX2: F: 5′‐AACCCCAAGATGCACAACTC‐3′,
R: 5′‐CGGGGCCGGTATTTATAATC‐3′;
CD44F: 5′‐ACCGACAGCACAGACAGAATC‐3′,
R: 5′‐GTTTGCTCCACCTTCTTGACTC‐3′;
GAPDH: F: 5′‐TGCACCACCAACTGCTTAG‐3′,
R: 5′‐GATGCAGGGATGATGTTC‐3′.

### 2.4 Lysis of cells and immunoblotting analysis

Cells were washed three times with cold phosphate‐buffered saline (PBS) and lysates were collected using cold radioimmunoprecipitation assay lysis buffer. Lysis buffer was supplemented with ethylenediaminetetraacetic acid (EDTA)‐free complete mini protease inhibitor cocktail (1 tablet per 10 mL lysis buffer) immediately before use. After incubating on ice for 15 minutes, lysates were centrifuged for 15 minutes at 18 000 rpm at 4°C. The supernatants were saved and protein concentrations were measured. Protein lysates were subjected to sodium dodecyl sulfate‐polyacrylamide gel electrophoresis (SDS‐PAGE) and immunoblotting (IB) analysis as described previously with slight modification.^30^ Samples were heated at 70°C for 15 minutes, instead of boiling for 5 minutes. SOX2 (3579S‐CST), NANOG (3580S‐CST), OCT4 (2750S‐CST), SNAIL (3879S‐CST), SLUG (9585S‐CST), CD44 (3570S‐CST), E‐cadherin (3195S‐CST), N‐cadherin (14215S‐CST), and nucleoporin (2598S‐CST) antibodies for IB analysis were purchased from Cell Signaling Technology, Inc (Danvers, MA). AR antibody (SC‐7305) was obtained from Santa Cruz Biotechnology (Santa Cruz, CA). Antibody to GAPDH (G9545) was purchased from Sigma‐Aldrich. Horseradish peroxidase–conjugated secondary antibodies were obtained from Kirkegaard & Perry Laboratories (Gaithersburg, MD; anti‐rabbit) and Santa Cruz Biotechnology (anti‐mouse). Protein estimation reagent kit, molecular weight protein standards, and polyacrylamide solutions were purchased from Bio‐Rad (Hercules, CA). Polyvinylidene fluoride membrane for IB analysis was obtained from Millipore Corp. (Bedford, MA), and ECL reagent was purchased from Pierce (Rockford, IL).

### 2.5 Human prostate lysates and IB analysis

Human prostate normal tissue lysates (normal; ab30304) and human prostate tumor tissue (TT) lysates (adenocarcinoma; ab30305) were purchased from Abcam (Cambridge, MA). Samples were heated at 70°C for 15 minutes and subjected to SDS‐PAGE and IB analyses with SOX2 and GAPDH antibodies.

### 2.6 Cytoplasmic and nuclear protein fraction preparation

Preparation of cytoplasmic and nuclear protein fractions was done as previously described.^30^ Briefly, a lysis buffer comprising of 10 mM Tris pH 7.9, 1.5 mM MgCl_2_, 10 mM KCl, 0.5 mM ethylene glycol‐bis(β‐aminoethyl ether)‐*N*,*N*,*N*′,*N*′′‐tetraacetic acid (EGTA), and protease inhibitor (1 tablet per 10 mL buffer) was used to lyse cells. Lysates were then centrifuged at 500*g* for 5 minutes at 4°C to separate the nuclear pellet from the supernatant. The supernatant, which constitutes the cytosolic component, was collected. The nuclear pellet was resuspended in nuclear lysis buffer containing 20 mM Tris pH 7.5, 25% glycerol, 1.5 mM MgCl_2_, 400 mM NaCl, and 0.5 mM EGTA. The suspension was centrifuged at 20 000*g* for 15 minutes at 4°C, and the supernatant comprising the nuclear component was collected. The lysates were analyzed by IB analysis as previously described.^35^

### 2.7 Immunostaining

SOX2 antibody (3579S‐CST), fluorochrome‐conjugated secondary antibody (Alexa Fluor 488, 4412‐CST) and mounting media with 4′,6‐diamidino‐2‐phenylindole (DAPI) were obtained from Cell Signaling Technology, Inc. PC3, LNCaP, and DU145 cells were cultured on coverslips in six‐well plates overnight at 37°C before staining.^30^ Cells were washed three times in PBS at room temperature (PBS‐RT) for 5 minutes each, and fixed in 4% paraformaldehyde‐PBS for 15 minutes. Cells were then blocked in blocking buffer containing 1× PBS/5% normal serum/0.3% Triton X‐100 for 1 hour. Subsequently, incubated with SOX2 antibody (1:100 dilution) in antibody dilution buffer containing 1× PBS/1% bovine serum albumin/0.3% Triton X‐100, overnight at 4°C. Cells were then washed three times in PBS‐RT for 5 minutes each, and incubated with the fluorochrome‐conjugated secondary antibody (1:1000 dilution) diluted in antibody dilution buffer for 3 hours at room temperature in the dark. Cells were then rinsed three times in PBS‐RT for 5 minutes each, mounted on slides using mounting media containing DAPI, and sealed with nail polish. The slides were viewed and photographed on Zeiss LSM 510 META Confocal Laser Scanning Microscopes (Zeiss, Germany). Images were analyzed with ImageJ software program, scriptable Java app for scientific image processing (Softonic International Ed. Media TIC, Barcelona, Spain).

### 2.8 KD of SOX2 in PC3 cells using small interfering RNA

PC3 cells were grown in six‐well plates overnight at 37°C, and allowed to reach around 60% confluency. Small interfering RNA (siRNA) designed against SOX2 (GE Healthcare Dharmacon Inc, Lafayette, CO) was transfected into PC3 cells using Lipofectamine 2000 (Thermo Fisher Scientific, Waltham, MA). SMARTpool siRNA represent four pooled SMART‐selected siRNA duplexes that target the SOX2 gene. Scrambled nontargeting RNA interference (RNAi) was used as the negative control (Thermo Fisher Scientific Inc, Waltham, MA). SOX2‐scrambled RNAi and siRNA were used to a final concentration of 100 nM. Five hours after the KD, the media in the plates were changed to complete RPMI media. Twenty‐four hours later, cell lysates were collected, and subjected to SDS‐PAGE and IB analysis to confirm the KD.

### 2.9 KD of AR in PCA2B cells using siRNA

PCa2b cells were grown in six‐well plates overnight at 37°C, and allowed to reach around 60% confluency. Human AR siRNA (Dharmacon, Horizon, Lafayette, CO) was transfected into PCa2b cells using Lipofectamine 2000. Scrambled nontargeting siRNA was used as the negative control (Thermo Fisher Scientific Inc.). AR scrambled RNAi and siRNA were used to a final concentration of 100 nM. Twenty‐four hours after the KD, the media in the plates were changed to complete RPMI media. Twenty‐four hours later, cell lysates were collected, and subjected to SDS‐PAGE and IB analysis to confirm KD and tumorsphere formation assay was performed.

### 2.10 Wound closure assay

Uniform vertical streaks were made in the monolayer culture with 200 μL pipette tips. The cells were immediately washed three times with RPMI medium with 10% FBS to remove detached cells. Mitomycin C (10 μg/mL; Sigma‐Aldrich) was added to the medium to inhibit proliferation, so that migration can be effectively followed.^3^ Mitomycin C is known to inhibit DNA synthesis using forming covalent crosslinks between complementary strands of DNA. This ultimately prevents separation of complementary strands of DNA, thereby inhibiting DNA replication. Cell migration was monitored for 24 hours, and pictures were taken at 0, 18, and 24 hours time points with a digital SPOT camera attached to an inverted Nikon phase contrast microscope (Nikon Inc., Melville, NY).

For wound closure assay in PC3 cells with KD of SOX2, siRNA KD of SOX2 was first performed on PC3 cells as described above. Twenty‐four hours post‐KD, the SOX2 siRNA transfected cells and nontargeting control siRNA transfected cells were replated in six‐well plates, grown overnight at 37°C, and allowed to reach near‐confluent levels. The wound closure assay was then performed as mentioned above. Parallel cultures were used to confirm SOX2 KD as compared with control siRNA‐treated cells

### 2.11 Tumorsphere formation assay

PCa cells that were 80% to 90% confluent were detached with trypsin‐EDTA solution. The cells were centrifuged for 3 minutes at 1200 rpm and the supernatant discarded. The cells were then resuspended in a small volume of StemXVivo‐Serum‐Free Tumorsphere Media (R&D Systems Inc., Minneapolis, MN) (CCM012) containing 2 U/mL heparin (2812; Tocris, Bio‐Techne Corporation, Minneapolis, MN) and 0.8 µg/mL hydrocortisone (4093; Tocris). The cells were plated at 0.05 × 10^6^ cells per well in a six‐well ultralow adhesion culture plate and cultured at 37°C and 5% CO_2_ for 7 days to induce tumorsphere formation as described.^36^, ^37^ After 7 days tumorspheres were imaged using a Cytation 3 Imaging Reader from Biotek (Winooski, VT).

### 2.12 Statistical analysis

All values presented as mean ± SEM. A value of *P* less than 0.05 was considered significant. Two‐tailed Student *t* test determined statistical significance. All of the data were analyzed with GraphPad Prism (GraphPad Software, Inc, La Jolla, CA).

## 3 RESULTS

### 3.1 PC3 cells demonstrate a marked increase in SOX2 expression at protein and messenger RNA levels as compared with other PCa cell lines

**Figure 1:**
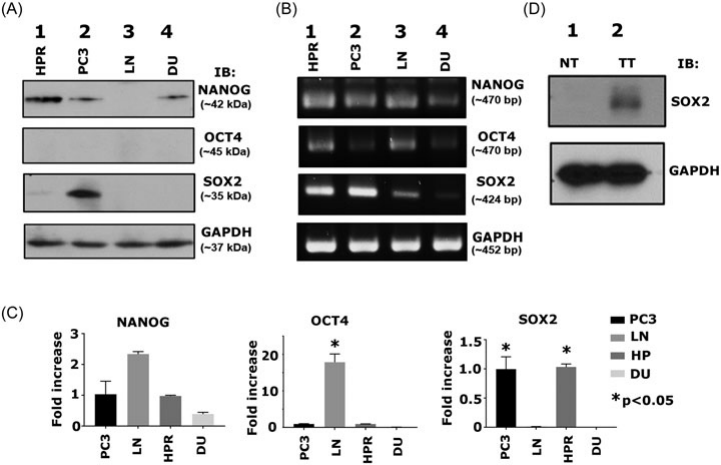
Characterizing the expression of stemness factors in PCa cell lines. A, IB analysis. An equal amount of protein lysates (40 μg) made from PC3 (lane 2), LNCaP (lane 3), DU145 (lane 4), and control HPR1 (lane 1) cells were immunoblotted with NANOG, OCT4, or SOX2 antibodies to detect total cellular levels of the respective proteins. B, RT‐PCR analysis of NANOG, OCT4, and SOX2 expression in PC3 (lane 2), LNCaP (lane 3), DU145 (lane 4), and control HPR1 (lane 1) cells. C, Real‐time PCR analysis of NANOG, OCT4, and SOX2 expression in PC3 (lane 1), LNCaP (lane 2), control HPR1 (lane 3), and DU145 (lane 4) cells. D, IB analysis of prostatic normal and tumor lysates is shown. Total cellular lysates from normal (lane 1) and prostatic tumor (lane 2) tissue (20 μg) were immunoblotted with SOX2. GAPDH was used as a loading control for IB, RT‐PCR, and real‐time PCR analysis (A‐D). The results represent one of the three separate experiments performed with the same results. OCT4: *P < 0.05 versus PC3, HPR, and DU; SOX2: *P < 0.05 versus LN and DU. GAPDH, glyceraldehyde 3‐phosphate dehydrogenase; IB, immunoblotting; NT, normal prostatic tissue; OCT4, octamer‐binding transcription factor 4; PCa, prostate cancer; RT‐PCR, reverse‐transcription polymerase chain reaction; SOX2, sex‐determining region Y (SRY)‐box 2; TT, tumor tissue

Here, our primary aim is to identify the expression of stemness factors at messenger RNA (mRNA) and protein levels in different PCa cell lines derived from human bone (PC3), brain (DU145), or lymph node (LNCaP) metastases. Normal prostatic epithelial cells (HPR1) were used as controls. IB, RT‐PCR, and real‐time PCR analysis were used to confirm the expression levels in the indicated cell lines. NANOG expression was more in PC3 and DU145 cells at the protein level (Figure 1A, lanes 2 and 4); however its expression was considerably lower than HPR1 cells (Figure 1A, lane 1). The expression of NANOG at mRNA levels was not significantly different in all the cell lines tested (Figure 1B and 1C). OCT4 is not expressed at the protein levels of all the cell lines tested (Figure 1A) but OCT4 mRNA levels were found to be more in LNCaP cells (Figure 1B, lane 3, and Figure 1C) as compared with PC3 and DU145 cells (Figure 1B, lanes 2 and 4, and Figure 1C). Interestingly, SOX2 expression was more in PC3 cells at the protein (Figure 1A, lane 2) and mRNA levels (Figure 1B, lane 2, and Figure 1C). SOX2 was not expressed in LNCaP and DU145 cells (Figure 1A, lanes 3 and 4). Furthermore, while there was more SOX2 expression at the mRNA level (Figure 1B, lane 1, and Figure 1C), the protein level was considerably lower in HPR1 cells (Figure 1A, lane 1). Since there was an increase in SOX2 in PC3 cells, we determined the expression levels of SOX2 protein in human prostatic TT lysates (Figure 1D, lane 2). SOX2 expression was more in TT lysates than the normal prostatic tissue (NT) lysates (Figure 1D, lane 1), suggesting that expression of SOX2 may have a role in the progression of cancer. However, further analyses are required to determine the expression levels of other stem cell markers in TT lysates. Since advanced PCa is known to metastasize to the bones in almost 85% to 100% of cases, we suggest that increased SOX2 could be a potential candidate to pursue in bone metastatic PC3 cells.

### 3.2 SOX2 localization is more in the nucleus of PC3 cells

**Figure 2:**
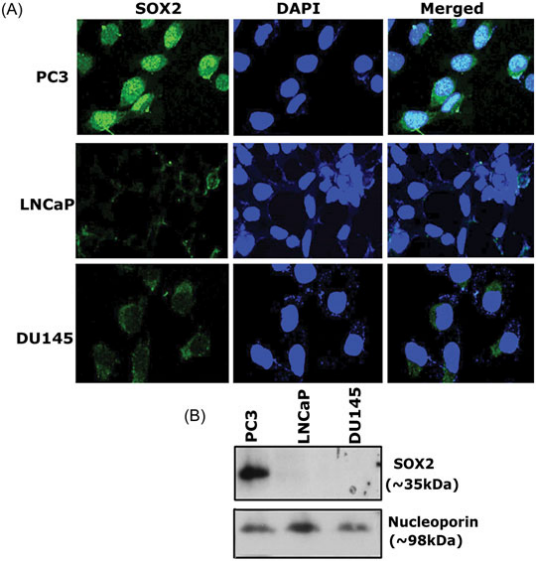
Analysis of the localization of SOX2 in prostate cancer cell lines. A, Immunostaining and confocal microscopy analysis of the distribution of SOX2 (green) in PC3, LNCaP, and DU145 cells. DAPI nuclear counterstain (blue) was used to demonstrate the specific localization of SOX2 in the nuclei of PC3 cells. B, Immunoblotting analyses of nuclear lysates from indicated cell lines with a SOX2 antibody validates the immunostaining analysis of the nuclear localization of SOX2 in PC3 cells (lane 1). Nucleoporin immunoblot demonstrates equal loading of nuclear proteins in each lane. The results represent one of the three separate experiments performed with similar results. DAPI, 4′,6‐diamidino‐2‐phenylindole; SOX2, sex‐determining region Y (SRY)‐box 2

Since SOX2 functions as a transcriptional factor, we proceeded to determine its localization by immunostaining and confocal analyses (Figure 2A). We have shown here that the localization of SOX2 was more in the nucleus of PC3 cells. It is nearly undetectable in LNCaP and DU145 cells. IB analysis of nuclear lysates from these cells indeed corroborates this observation (Figure 2B).

### 3.3 KD of SOX2 reduces expression of EMT‐related factors such as SNAIL and SLUG in PC3 cells

**Figure 3:**
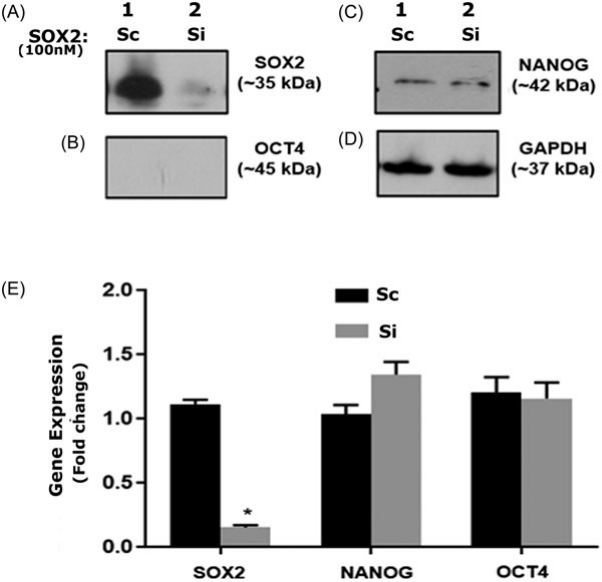
Analysis of the effect of SOX2 KD on other stemness factors in PC3 cells (A‐D). IB analyses with indicated antibodies. Protein lysates (40 μg) made from PC3 cells transfected with a scrambled RNAi (Sc; lane 1) and siRNA (Si, lane 2) to SOX2 were used for IB analyses. A, The immunoblot in (A) confirms the KD of SOX2 in siRNA‐treated cells. B‐D, SOX2 KD effects on the expression of OCT4 and NANOG were determined using the respective antibody. The blot was stripped three times sequentially and blotted with an antibody to OCT4 (B), NANOG (C), and GAPDH (D). GAPDH immunoblot demonstrates equal loading of proteins in each lane and there was no loss of signal due to stripping. The results shown are representative of three independent experiments. E, Real‐time PCR analysis. The expression levels of SOX2, NANOG, and OCT4 mRNA upon SOX2 KD was determined by real‐time PCR analysis and normalized relative to GAPDH expression. Bar represents the mean ± SEM of three different experiments, *P < 0.05 vs scrambled (Sc) RNAi‐treated cells. GAPDH, glyceraldehyde 3‐phosphate dehydrogenase; IB, immunoblotting; KD, knockdown; mRNA, messenger RNA; OCT4, octamer‐binding transcription factor 4; PCR, polymerase chain reaction; siRNA, small interfering RNA; RNAi, RNA interference; SOX2, sex‐determining region Y (SRY)‐box 2

To determine the functional role of SOX2, we used the KD strategy. Here, our goal is to determine the following: (a) Is there any compensatory mechanism(s) by other stemness factors, (b) Does SOX2 regulate the expression of any EMT markers, and (c) Does it have a role in the migration and tumorsphere formation of PC3 cells. SMARTpool siRNA to SOX2 was used to KD SOX2 expression in PC3 cells and the KD was confirmed by IB and real‐time PCR analyses (Figure 3A and 3E).

**Figure 4:**
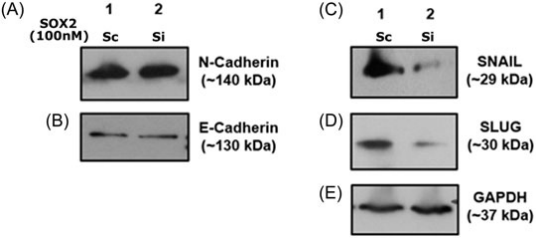
The effect of SOX2 knockdown on the expression of EMT‐related factors. An equal amount of protein lysates (40 μg) made from PC3 cells transfected with scrambled RNAi (Sc; lane 1) and siRNA (Si; lane 2) targeting SOX2 were used for immunoblotting analyses (A‐E). The N‐cadherin blot in (A) was stripped and reprobed with an E‐cadherin antibody (B). The SNAIL blot in (C) was stripped twice sequentially and blotted with a SLUG (D) and GAPDH (E) antibody. GAPDH immunoblot demonstrates equal loading of proteins in each lane. Data represent one of the three independent experiments with similar results. EMT, epithelial‐mesenchymal transition; GAPDH, glyceraldehyde 3‐phosphate dehydrogenase; RNAi, RNA interference; siRNA, small interfering RNA; SOX2, sex‐determining region Y (SRY)‐box 2

**Figure 5:**
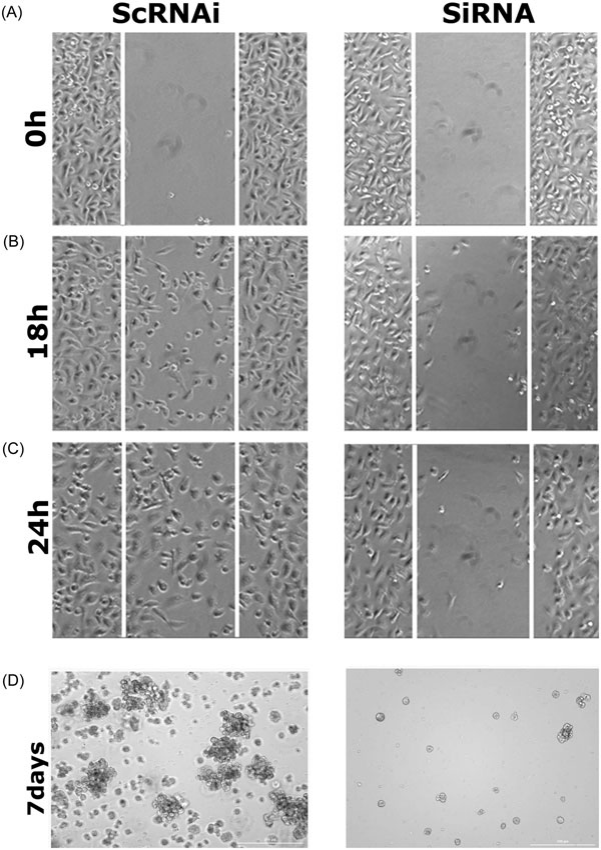
The effect of SOX2 silencing on the migration and the tumorsphere formation in PC3 cells. PC3 cells transfected with scrambled (Sc) RNAi and siRNA to SOX2 were subjected to wound closure and tumorsphere formation assays. A‐C, Phase contrast micrographs show migration at 0, 18, and 24 hours. D, Cell imaging in the multimode microscope (cytation 3) shows tumorsphere formation in indicated PC3 cells. Scale bar: 200 µm. The results shown are representative of three independent experiments. RNAi, RNA interference; siRNA, small interfering RNA; SOX2, sex‐determining region Y (SRY)‐box 2

(1) Other stemness factors do not compensate loss of SOX2. OCT4 is not expressed at the protein level in PC3 cells (Figure 1A, lane 2), and its expression pattern did not change with the introduction of SOX2‐scrambled RNAi or siRNA (Figure 3B). Similarly, the expression level of NANOG is not affected in these cells (Figure 3C). At mRNA level, the loss of SOX2 does not significantly alter expression of the other stemness factors (Figure 3E). These results confirm that the loss of SOX2 is not compensated by the upregulation of other stemness factors of the core embryonic transcription factor machinery, thereby reinforcing the relevance of the KD strategy used to elucidate the functional role of SOX2.

(2) Loss of SOX2 has an impact on the expression of EMT regulatory factors such as SNAIL and SLUG but not EMT markers. We first assessed the expression of known EMT markers such as E‐ and N‐cadherin by IB analysis in SOX2‐scrambled RNAi and siRNA‐treated cells. No significant changes in the expression of E‐ and N‐cadherin were observed in SOX2‐scrambled RNAi and siRNA‐treated PC3 cells (Figure 4A and 4B). IB analysis indeed revealed that SOX2 KD reduces the expression of SNAIL and SLUG (Figure 4C and 4D). These observations suggest that SOX2 does not have any direct effect on the expression of E‐ or N‐cadherin.

(3) SOX2 KD reduces cell migration and tumorsphere formation. First we sought to determine whether SOX2 influences migration. SOX2‐scrambled RNAi and siRNA‐treated PC3 cells were subjected to wound closure assay. Wound closure was monitored for 18 hours (Figure 5B) and 24 hours (Figure 5C). Scrambled RNAi–treated cells displayed greater migration and wound closure capabilities (Figure 5B and 5C, left panel), with the wound almost closing up at 24 hours (Figure 5C, left panel). A significant decrease in the migration was observed at 18 and 24 hours in PC3 cells transfected with the siRNA (Figure 5B and 5C, right panel). Cells pretreated with mitomycin C ensures that the observed results on cell migration are independent of cell proliferation.

Secondly, we wanted to determine whether SOX2 influences tumorsphere formation. CSCs can form tumorspheres in vitro when plated in limited numbers and under serum‐free condition supplemented with growth factors. The cells in a tumorsphere formation assay appear fused together and they are indicative of the cancer stem/progenitor cell population within the culture.^36^, ^37^ Scrambled RNAi and siRNA‐treated PC3 cells were subjected to in vitro tumorsphere formation. Scrambled RNAi–treated cells displayed more tumorspheres (Figure 5D, left panel) in comparison with SOX2 siRNA–treated PC3 cells (Figure 5D, right panel) as seen by the formation of spheroids that are fused together to form a solid cluster of cells. A significant decrease in wound closure and tumorsphere formation in PC3 cells KD of SOX2 suggests that SOX2 may play an important role in cell migration, self‐renewal, and differentiation (Figure 5A and 5D), which is a part of the EMT process.

### 3.4 CD44 has a potential role in the expression of SOX2 and migration of PC3 cells

**Figure 6:**
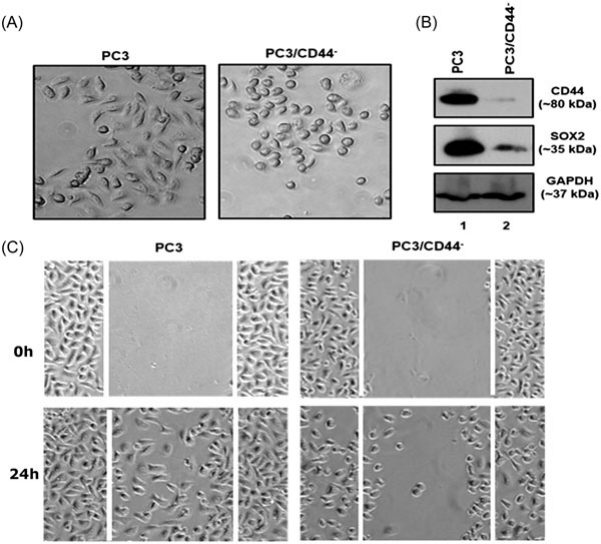
Analysis of effects of CD44 knockdown on cell morphology, SOX2 expression, and cell migration. A, Phase contrast micrograph shows the morphology of PC3 and PC3/CD44^−^ cells at ×100 magnification. B, An equal amount of protein lysates (40 μg) made from PC3 (lane 1) and PC3/CD44^−^ (lane 2) cells were used for IB analyses. IB was done with an antibody to CD44 to confirm the knockdown (top panel). The blot was stripped twice sequentially and blotted with an antibody to SOX2 and GAPDH. GAPDH immunoblot demonstrates equal loading of proteins in each lane (bottom panel) and there was no loss of signal during stripping. C, Wound closure assay. Phase contrast micrographs show the migration of PC3 and PC3/CD44^−^ cells at 0 and 24 hours. The results shown are representative of three independent experiments. CD44, cluster of differentiation 44; GAPDH, glyceraldehyde 3‐phosphate dehydrogenase; IB, immunoblotting; SOX2, sex‐determining region Y (SRY)‐box 2

We have previously shown that CD44 increases the invasive property of PC3 cells.^30^ Here, we used PC3 cells and PC3 cells stably KD of CD44 (PC3/CD44^−^) to examine SOX2 expression levels. As shown previously,^30^ PC3/CD44^−^ cells were more rounded and less adhesive as compared with PC3 cells (Figure 6A). CD44 KD in PC3 cells resulted in significant downregulation of SOX2 expression (Figure 6B, lane 2). Loss of cell adhesion corresponds with a decreased migration in PC3/CD44^−^ cells (Figure 6C). These observations further highlight the ability of CD44 to influence SOX2 expression, and ultimately regulate the migration. SOX2 may be a potential downstream target of CD44, having an important role in cell migration.

### 3.5 Expression of AR reduces SOX2 and CD44 expression, and cell migration in PC3 cells

Androgen/AR axis has been shown to modulate stemness characteristics.^5^, ^38^, ^39^ PC3 cells are AR^−^ cells. Our aim here is to determine the following: (a) influence of AR on the expression of SOX2 and CD44, and (b) its impact on cell migration. In exploring the AR‐CD44‐SOX2 axis, we used PC3 cells (negative for AR), and PC3 cells stably expressing AR (PC3/AR^+^).

**Figure 7:**
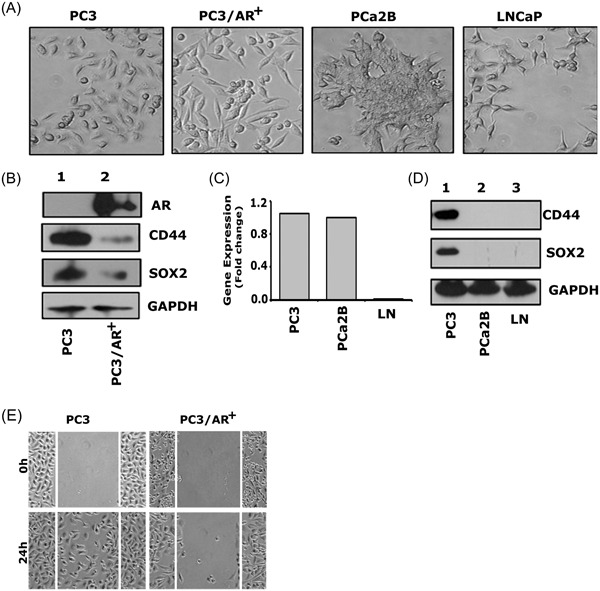
Analysis of the effects of AR expression in PC3 cells on cell morphology, CD44 and SOX2 expression, and cell migration. A, Phase contrast micrograph shows the morphology of PC3, PC3/AR^+^, PCa2B, and LNCaP cells at ×100 magnification. PCa2b cells grow in clumps in culture and all the other cell lines have elongated morphology with spindle‐like projections. B, IB analysis of lysates (40 μg protein) from PC3 (lane 1) and PC3/AR^+^ (lane 2) cells with an antibody to AR (~110 kDa), CD44 (~80 kDa), SOX2 (~35 kDa), and GAPDH (loading control; ~37 kDa) is shown. AR and SOX2 immunoblots were stripped and immunoblotted with an antibody to CD44 and GAPDH, respectively. IB analysis with an antibody AR confirms the expression of AR in PC3/AR^+^ cells. C, Real‐time PCR analysis of CD44 expression in indicated cell lines. The expression was normalized relative to GAPDH expression. D, Immunoblotting analyses in AR^−^ (PC3) and AR^+^ (PCa2B and LNCaP) cells with antibodies to CD44 and SOX2. GAPDH immunoblot demonstrates equal loading of proteins in each lane (B, D). E, Analysis of the effect of AR expression in PC3 cells on migration using a wound closure assay. Phase contrast micrographs show the migration of PC3 and PC3/AR^+^ cells at 0 and 24 hours. Results represent one of three experiments performed. AR, androgen receptor; CD44, cluster of differentiation 44; GAPDH, glyceraldehyde 3‐phosphate dehydrogenase; PCR, polymerase chain reaction; SOX2, sex‐determining region Y (SRY)‐box 2

We used phase contrast microscopy to assess morphological changes in indicated PCa cell lines. PC3/AR^+^ cells are highly adhesive and have an elongated morphology with spindle‐like long projections (Figure 7A). Both PCa2b and LNCaP cells are androgen‐sensitive cells. PCa2b and LNCaP cells are positive for ARs. PCa2b cells are derived from bone metastasis of African American patient. Phase contrast microscopy analysis demonstrates that PCa2b cells grow in clumps in culture and LNCaP cells have an elongated morphology with spindle‐like projections (Figure 7A). AR expression in PC3 cells resulted in reduced levels of CD44 and SOX2 (Figure 7B, lane 2). Notably, the CD44 and SOX2 expression profile in PCa2b and LNCaP cells are very similar to that of PC3/AR^+^ cells (Figure 7C and 7D). Expression of CD44 and SOX2 are very negligible in these cells (Figure 7D, lanes 2 and 3). Although real‐time PCR analysis demonstrates the expression of CD44 at mRNA levels (Figure 7C) in PCa2b, the expression of the CD44 protein is repressed (Figure 7D, lane 2). The direct impact of AR on the inhibition of CD44 protein expression remains unclear and needs further elucidation.

We next examined the relevance of AR‐CD44‐SOX2 axis on cell migration, using the wound closure assay. PC3 cells and PC3/AR^+^ cells were subjected to this assay, and wound closure was monitored for 24 hours. PC3/AR^+^ cells displayed lower migration capabilities as compared with PC3 cells (Figure 7E). These results suggest that AR might impact the cell migration process via modulating CD44 and SOX2 expression.

### 3.6 KD of AR reduces CD44/SOX2 expression and tumorsphere formation in Pca2B cells

**Figure 8:**
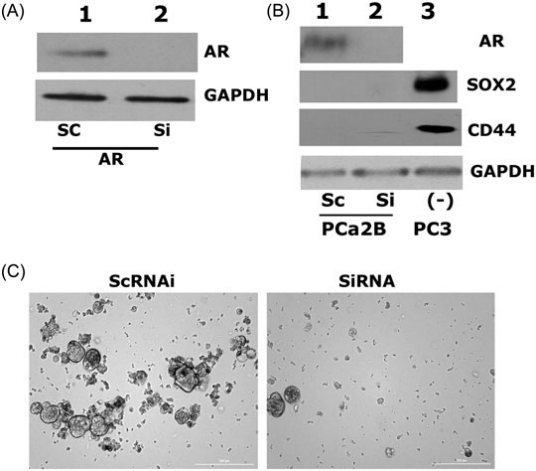
Elucidating the impact of AR silencing on tumorsphere formation in PCa2b cells. PCa2b cells transfected with scrambled RNAi (Sc) and siRNA (Si) were used for immunoblotting analyses (A, B) and tumorsphere formation (C). A, IB analysis with an AR (~110 kDa) and GAPDH (loading control; ~37 kDa) antibody. B, IB analyses with AR, SOX2, CD44, and GAPDH (loading control) antibody. IB with an AR antibody confirms the knockdown of AR (B, top) in PCa2b cells. Subsequently, same lysates proteins were used for the SOX2 (~35 kDa) and CD44 (~80 kDa) IB analyses which were done together. The blot was stripped and reprobed with a GAPDH antibody. C, Tumorsphere formation assay. Cell imaging multimode microscope (cytation 3) show tumorsphere formation in PCa2b cells. Scale bar: 200 µm. AR, androgen receptor; CD44, cluster of differentiation 44; GAPDH, glyceraldehyde 3‐phosphate dehydrogenase; SOX2, sex‐determining region Y (SRY)‐box 2

We then proceeded to determine whether KD of AR in PCa2b increases the expression of CD44 and SOX2. We performed Western blot analysis to confirm KD of AR in PCa2b cells (Figure 8A, lane 2). KD of AR in PCa2b did not upregulate either CD44 or SOX2 proteins (Figure 8B, lane 2). Lysates made from PC3 cells were used as a control (Figure 8B, lane 3). However, KD of AR in PCa2b resulted in decreased ability to form tumorspheres as compared with Scrambled RNAi–treated cells (Figure 8C). Taken together these results suggest that tumorsphere formation occur independently of CD44‐SOX2 signaling in PCa2b cells. Overexpression of AR in PC3 cells suppressed CD44 and SOX2 protein levels (Figure 7). AR may have a role in the repression of expression of CD44 in LNCaP and PCa2b cells. One would expect that KD of AR in PCa2b would increase CD44 and SOX2 expression. However AR KD did not influence the expression of CD44 and SOX2 proteins although CD44 is expressed at the mRNA level in PCa2b (Figure 7) and LNCaP cells.^40^ Expression of CD44 seems cell type‐specific and possibly depends on the unique physiological and pathological features of the metastatic organs. Together, these results raise several new questions on AR‐CD44‐SOX2 signaling axis which need further investigation.

## 4 DISCUSSION

While AR signaling is critical for the normal development, function, and homeostasis of the prostate gland, it is also thought to play a major role in PCa pathogenesis.^41^, ^42^ There is accumulating evidence suggesting that the androgen‐deprivation therapy initial therapeutic approach aimed at modulating AR signaling, results in the expansion of CSCs.^26^, ^43^, ^44^ These CSCs which survive through conventional therapies are thought to contribute toward treatment failure and tumor recurrence—clinical challenges associated with PCa therapy. The current study was performed to evaluate the expression of SOX2, OCT4, and NANOG transcription factors in PCa cell lines derived from different metastases, to identify the key stemness factor that determines the “molecular signature” of potential prostate CSCs. Moreover, we also wanted to examine the ability of AR to mediate stemness characteristics of PCa cells.

We first screened three cell lines from different metastases (PC3, DU145, and LNCaP) for stemness factor expression. LNCaP cells from lymph node metastasis are positive for AR, hormone‐sensitive, and have low‐metastatic potential. PC3 and DU145 cells from bone and brain metastasis, respectively, are negative for AR, hormone refractory, and have higher metastatic and malignant properties. Normal prostatic epithelial (HPR1) cells were used as controls.^31^ IB and RT‐PCR analyses revealed a markedly increased SOX2 expression at both protein and mRNA levels in PC3 cells. These results are consistent with previous studies by others that indicate that SOX2 was significantly increased in more malignant cell lines.^45^, ^46^ Moreover, the lack of substantial expression of OCT4 and NANOG in SOX2‐expressing PC3 cells suggest that SOX2 could potentially have a novel and significant role in metastasis, independent of its association with other stemness factors. Our strategy to explore the role of SOX2 as a putative marker of prostate CSCs was further driven by the results of our IB analysis of human prostate tissue samples, whereby increased SOX2 expression was observed in TTs as compared with normal tissues. Previous reports corroborate these observations, where SOX2 was found to be associated with tumorigenesis and its overexpression correlated with higher histologic grade and Gleason score.^45^ These findings reinforce the clinical relevance of SOX2 expression and its potential role in PCa progression. We decided to use PC3 cell line for all further experimental manipulation as it has the highest CD44 and SOX2 expression, and advanced PCa is known to metastasize to the bones.^47^, ^48^

Nuclear localization of the SOX2 protein in PC3 cells suggests that it may function as a key transcriptional regulator in these cells. Bearing in mind the accumulating evidence emphasizing the existence of shared molecular characteristics between CSCs and EMT cells, we used a SOX2 KD strategy to elucidate its functional role and potential downstream targets, with regard to EMT and cell migration. The prospect of a potential SOX2‐EMT axis would not be surprising, considering that this axis is known to have central roles in the invasion and metastasis of several human cancers.^24^, ^49^, ^50^ For instance, SOX2 silencing in colorectal cancer cells induced mesenchymal‐epithelial transition, a reciprocal process to EMT, with marked changes in the expression of key drivers of EMT such as SNAIL.^24^ Consistent with these observations, our analyses indeed revealed reduced expression of EMT regulatory factors SNAIL and SLUG with SOX2 silencing. These results corroborate the decrease in cell migration observed with the SOX2 KD. Our results suggest that SOX2 may have an important role in inducing cell migration, an EMT process, acting though SNAIL and SLUG downstream targets. However, the underlying molecular mechanism by which SOX2 regulates SNAIL and SLUG expression need further elucidation.

CD44 signaling has been shown to modulate the tumor microenvironment and promote EMT‐related events through its involvement in processes like cell trafficking, lymph node homing, cell‐ECM adhesion, and coordination of cytokine signaling.^51^ Strikingly, there is also growing evidence projecting CD44 to be a marker of CSCs in the breast, prostate, pancreas, ovarian, and colorectal cancers.^52^, ^53^ While CD44 has been thought to have a strategic role in PCa progression, the exact mechanism involved is still largely unknown. Hence, in the current study we examined the potential role of CD44, and its relationship with SOX2 using a KD approach. Our studies indeed reveal the ability of CD44 to modulate stemness characteristics of PCa cells, whereby the loss of CD44 reduced expression of SOX2. These observations are in line with reports in breast cancer, where they identified CD44, in particular, its intracellular domain to be critical in influencing the expression of stemness factors and the maintenance of breast CSCs.^29^ The morphological changes and a decrease in cell migration witnessed in CD44‐silenced cells further validate its role in the EMT process. Intriguingly, our results establish the presence of a reciprocal relationship between CD44 and SOX2. One likely explanation for CD44‐regulated SOX2 expression in PC3 cells may be the vision of SOX2 being a potential downstream target of CD44. Additional investigations are required to understand the prospective association between CD44 and SOX2 further.

Suppressing AR signaling remains the primary focus of therapeutic strategies for advanced PCa. However, despite initial favorable response this approach eventually leads to more aggressive disease. With the exact mechanism for treatment failure and tumor recurrence still unclear, some potential explanations put forward include the acquisition of CSC phenotype and EMT following selection pressures imposed by androgen‐deprivation therapy. The current study was undertaken to determine the influence of AR on the expression of SOX2 and CD44, and to examine the functional consequence of AR‐CD44‐SOX2 axis. The experimental strategy used involves the expression of AR in PC3 cells that are AR^−^. Our analyses revealed a significant decrease in the expression of CD44 and SOX2 in response to AR expression in PC3 cells, suggesting that AR signaling may have the potential to reduce stemness characteristics of these cells. Our results are in agreement with others that AR represses the expression of SOX2 and CD44.^30^, ^54^ Strikingly, with the introduction of AR in androgen‐independent PC3 cells begin to exhibit characteristics of androgen‐dependent PCa2b cells and LNCaP cells, regarding SOX2‐CD44 expression profiles. It is vital to note that both PC3 and PCa2b cells are derived from human bone metastasis, with AR being one of the key differentiating factors between them. However, KD of AR in PCa2b cells did not affect the expression of CD44 or SOX2. One explanation for this observation could be that PCa2b cells may preserve both prostate‐specific antigen (PSA) and androgen sensitivity in contrast to PC3 cells which do not express AR and PSA.^32^ The loss of CD44 expression could be compensated for by the secretion of PSA and androgen sensitivity. Further studies are needed to understand the molecular mechanism involved in the repression of CD44 in AR^+^ cells or the induction of CD44 expression in AR^−^ cells.

**Figure 9:**
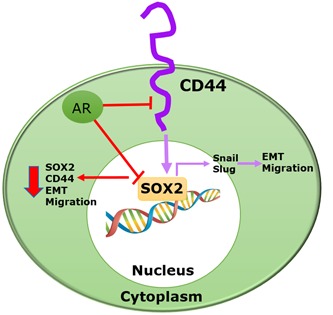
A schematic diagram illustrating the proposed mechanism of AR‐CD44‐SOX2 signaling. Purple highlights CD44‐SOX2 pathway leading to EMT and hence migration. Red highlights AR expression downregulates not only the expression of CD44 and SOX2 but also the processes of EMT and migration in prostate cancer PC3 cells. AR, androgen receptor; CD44, cluster of differentiation 44; EMT, epithelial‐mesenchymal transition; SOX2, sex‐determining region Y (SRY)‐box 2

Bone metastatic PCa PC3 cells that are AR^−^ highly express CD44 and SOX2. Expression of CD44 and SOX2 results in the upregulation of transcription factors SNAIL and SLUG, which leads to EMT and hence migration. SNAIL and SLUG have been shown to increase the migration of breast cancer cells.^55^ SOX2 KD reduces the levels of SNAIL and SLUG proteins but not cell junction molecules E‐ or N‐cadherin; however decreases cell migration and tumorsphere formation. Our observations in PCa PC3 cells substantiate the possible role of the CD44‐SOX2‐SNAIL/SLUG axis in migration and tumor progression. Forced expression of AR in PC3 cells results in downregulation of CD44 and SOX2 thereby preventing EMT and migration. Re‐expression of AR in PC3 cells may have a potential to reduce the stemness characteristics of these cancer cells (Figure 9). Reduction in stemness characteristics witnessed with AR expression correlated with decreased migration in PC3 cells. Our current studies focus on identifying the molecular mechanisms involved in the regulation of SOX2 by CD44 signaling and the role of CD44‐SOX2‐SNAIL/SLUG axis in eliciting EMT and migration/tumor progression.

## 5 CONCLUSIONS

Taken together, the results of our study would have significant clinical implications regarding enhancing our understanding of prostate CSCs. Here, we show the presence of an AR‐CD44‐SOX2 axis and recognize SOX2 as a putative CSC marker that can define the “molecular signature” of PCa stem cells. Our observations provide critical knowledge that would enable us to work toward designing a more comprehensive therapeutic approach for advanced PCa. SOX2 could be a potential therapeutic target to impede the progression of SOX2‐positive cancer cells or recurrence of androgen‐independent PCa.

## CONFLICTS OF INTEREST

The authors declare that there are no conflicts of interest.

## AUTHORS’ CONTRIBUTIONS

DS and LS participated in the design of the study, performed the experiments, analyzed the data, and wrote the manuscript. LS and SM participated in the preparation of lysates from different PCa cells and tumorsphere formation. RBF generated stable PC3 cell line expressing androgen receptor (PC3/AR^+^). MAC conceived and designed the study, analyzed the data with DS, and was involved in manuscript preparation. All authors read and approved the final manuscript.
